# Bis[*N*′-(3-cyano­benzyl­idene)isonicotino­hydrazide-κ*N*]silver(I) trifluoro­methane­sulfonate

**DOI:** 10.1107/S1600536809039579

**Published:** 2009-10-03

**Authors:** Cao-Yuan Niu, Hai-Yan Zhang, Xin-Sheng Wan

**Affiliations:** a College of Sciences, Henan Agricultural University, Zhengzhou 450002, People’s Republic of China

## Abstract

In the title compound, [Ag(C_14_H_10_N_4_O)_2_]CF_3_SO_3_, two N atoms from two independent pyridyl rings of two *N*′-3-cyano­benzyl­ideneisonicotinohydrazide ligands coordinate to the unique Ag^I^ ion, forming a nearly linear coordination geometry. Adjacent silver complexes are primarily linked together by Ag⋯N inter­actions, with Ag⋯N separations of 2.877 (2) and 3.314 (2) Å. On the other hand, one CF_3_SO_3_^−^ anion inter­acts with hydrazone groups of two neighbouring ligands *via* N—H⋯O hydrogen bonds. These weak inter­molecular inter­actions contribute to the formation of supra­molecular chains. In addition, there are Ag⋯O inter­actions [2.787 (2) Å] between Ag and O atoms from adjacent chains.

## Related literature

For the coordination of silver ions and properties of silver coordination compounds, see: Dong *et al.* (2004 ▶); Niu *et al.* (2008 ▶, 2009 ▶); Sumby & Hardie (2005 ▶); Abu-Youssef *et al.* (2007 ▶).
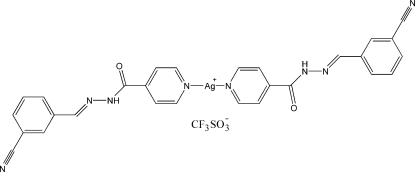


## Experimental

### 

#### Crystal data

[Ag(C_14_H_10_N_4_O)_2_]CF_3_SO_3_
*M* *_r_* = 757.46
Triclinic, 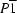

*a* = 7.5481 (17) Å
*b* = 14.164 (3) Å
*c* = 14.175 (3) Å
α = 87.895 (4)°
β = 89.918 (4)°
γ = 81.355 (4)°
*V* = 1497.2 (6) Å^3^
*Z* = 2
Mo *K*α radiation
μ = 0.82 mm^−1^
*T* = 173 K
0.32 × 0.22 × 0.17 mm

#### Data collection

Bruker APEXII CCD area-detector diffractometer
Absorption correction: multi-scan (*SADABS*; Bruker, 2005 ▶) *T*_min_ = 0.780, *T*_max_ = 0.874
8251 measured reflections
5461 independent reflections
3857 reflections with *I* > 2σ(*I*)
*R*_int_ = 0.023

#### Refinement

*R*[*F* ^2^ > 2σ(*F* ^2^)] = 0.060
*wR*(*F* ^2^) = 0.175
*S* = 1.03
5461 reflections
433 parameters
H atoms treated by a mixture of independent and constrained refinement
Δρ_max_ = 1.04 e Å^−3^
Δρ_min_ = −0.87 e Å^−3^

### 

Data collection: *APEX2* (Bruker, 2005 ▶); cell refinement: *SAINT* (Bruker, 2005 ▶); data reduction: *SAINT*; program(s) used to solve structure: *SHELXS97* (Sheldrick, 2008 ▶); program(s) used to refine structure: *SHELXL97* (Sheldrick, 2008 ▶); molecular graphics: *SHELXL97* and *DIAMOND* (Brandenburg, 2005 ▶); software used to prepare material for publication: *SHELXL97*.

## Supplementary Material

Crystal structure: contains datablocks I, global. DOI: 10.1107/S1600536809039579/bh2248sup1.cif

Structure factors: contains datablocks I. DOI: 10.1107/S1600536809039579/bh2248Isup2.hkl

Additional supplementary materials:  crystallographic information; 3D view; checkCIF report

## Figures and Tables

**Table:** 

Ag1—N5	2.160 (4)
Ag1—N1	2.169 (4)

**Table:** 

N5—Ag1—N1	172.56 (17)

**Table 2:** Hydrogen-bond geometry (Å, °)

*D*—H⋯*A*	*D*—H	H⋯*A*	*D*⋯*A*	*D*—H⋯*A*
N2—H29⋯O5^i^	0.86 (6)	2.27 (6)	3.125 (9)	173 (5)
N6—H28⋯O3^ii^	0.90 (5)	2.12 (6)	2.982 (7)	161 (5)

Symmetry codes: (i) 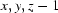; (ii) 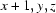.

**Fig. 1.**
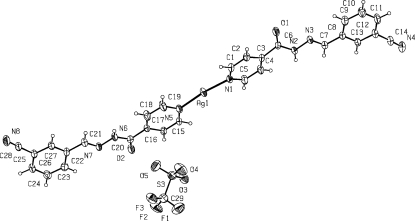
A view of the AgI coordination environment in the monomeric structure of (I), showing the atom-labelling scheme. Displacement ellipsoids are drawn at the 30% probability level.

**Fig. 2.**
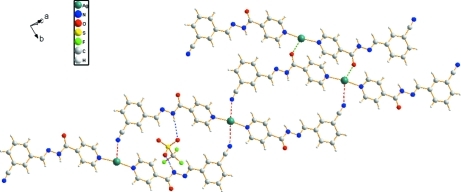
Packing diagram showing three intermolecular interactions: hydrogen bonds are indicated by blue dashed lines, Ag···N interactions by red dashed lines, and Ag···O interactions by green dashed lines.

## supplementary crystallographic information

### Comment

In the title compound, (I), the silver(I) ion is coordinated by two nitrogen atoms from two independent pyridyl rings of two different ligands, forming a slightly distorted linear coordination geometry (Fig. 1). Related bond distances and angle around the metal center are shown in Table 1.

There are N—H···O hydrogen bonds between hydrazone groups from 3-cyanobenzylidene isonicotinohydrazide and counteranions CF_3_SO_3_^-^ (Table 2). Besides, there are weak Ag···N interactions between two neighbouring silver complexes with separations of 2.877 (2) and 3.314 (2) Å. Hydrogen bonds and Ag···N interactions link parallel silver monomers together to construct interesting supramolecular one-dimensional chains. Furthermore, two adjacent supramolecular one-dimensional chains are linked together *via* Ag···O interactions, with the separation of 2.787 (2) Å (Fig. 2). All these intermolecular interactions have the contribution to the three-dimensional structure of the title compound.

It is noteworthy that the coordination geometry of the silver metal center can be affected by many factors, such as coordination natures of organic ligands, temperature, counteranions, *etc.* (Dong *et al.*, 2004; Niu *et al.*, 2009; Sumby & Hardie, 2005; Abu-Youssef *et al.*, 2007). We have reported a Ag(I) polymeric structure recently (Niu *et al.*, 2008), which includes a ligand isomeric to the one used in this paper (3-cyanobenzylidene isonicotinohydrazide). It showed that the position of the CN functional group seems to have a great influence on the structures of the resulting compounds (monomeric *versus* polymeric).

### Experimental

A solution of AgCF_3_SO_3_ (0.026 g, 0.1 mmol) in CH_3_OH (10 ml) was carefully layered on a CH_3_OH/CHCl_3_ solution (5 ml/10 ml) of 3-cyanobenzylidene isonicotinohydrazide (0.025 g, 0.1 mmol) in a straight glass tube. About ten days later, colourless single crystals suitable for X-ray analysis were obtained.

### Refinement

C-bound H atoms were placed in calculated positions and refined using a riding model [C—H = 0.95 Å and *U*_iso_(H) = 1.2*U*_eq_(C)]. The N-bound H atoms were first introduced in calculated positions and refined freely with *U*_iso_(H) = 1.2*U*_eq_(carrier N). The final difference Fourier map had a highest peak at 0.76 Å from atom H24 and a deepest hole at 0.64 Å from atom S3, but was otherwise featureless.

### Crystal data

**Table:**

[Ag(C_14_H_10_N_4_O)_2_]CF_3_SO_3_	*Z* = 2
*M**_r_* = 757.46	*F*(000) = 760
Triclinic, *P*1	*D*_x_ = 1.680 Mg m^−^^3^
Hall symbol: -P 1	Mo *K*α radiation, λ = 0.71073 Å
*a* = 7.5481 (17) Å	Cell parameters from 2331 reflections
*b* = 14.164 (3) Å	θ = 2.0–25.5°
*c* = 14.175 (3) Å	µ = 0.82 mm^−^^1^
α = 87.895 (4)°	*T* = 173 K
β = 89.918 (4)°	Needle, yellow
γ = 81.355 (4)°	0.32 × 0.22 × 0.17 mm
*V* = 1497.2 (6) Å^3^	

### Data collection

**Table:**

Bruker APEXII CCD area-detector diffractometer	5461 independent reflections
Radiation source: fine-focus sealed tube	3857 reflections with *I* > 2σ(*I*)
graphite	*R*_int_ = 0.023
φ and ω scans	θ_max_ = 25.5°, θ_min_ = 2.0°
Absorption correction: multi-scan (*SADABS*; Bruker, 2005)	*h* = −8→9
*T*_min_ = 0.780, *T*_max_ = 0.874	*k* = −15→17
8251 measured reflections	*l* = −15→17

### Refinement

**Table:**

Refinement on *F*^2^	Primary atom site location: structure-invariant direct methods
Least-squares matrix: full	Secondary atom site location: difference Fourier map
*R*[*F*^2^ > 2σ(*F*^2^)] = 0.060	Hydrogen site location: inferred from neighbouring sites
*wR*(*F*^2^) = 0.175	H atoms treated by a mixture of independent and constrained refinement
*S* = 1.03	*w* = 1/[σ^2^(*F*_o_^2^) + (0.0934*P*)^2^ + 1.7184*P*] where *P* = (*F*_o_^2^ + 2*F*_c_^2^)/3
5461 reflections	(Δ/σ)_max_ < 0.001
433 parameters	Δρ_max_ = 1.04 e Å^−^^3^
0 restraints	Δρ_min_ = −0.87 e Å^−^^3^
0 constraints	

### Fractional atomic coordinates and isotropic or equivalent isotropic displacement parameters (Å^2^)

**Table:**

	*x*	*y*	*z*	*U*_iso_*/*U*_eq_	
Ag1	0.93777 (7)	0.21340 (3)	0.17729 (3)	0.0685 (2)	
N1	0.8202 (6)	0.1314 (3)	0.0743 (3)	0.0514 (11)	
N2	0.5612 (6)	−0.0094 (4)	−0.2091 (3)	0.0513 (11)	
N3	0.5110 (6)	−0.0664 (3)	−0.2783 (3)	0.0521 (11)	
N4	0.0251 (8)	0.0449 (4)	−0.7023 (4)	0.0721 (14)	
N5	1.0223 (6)	0.3075 (3)	0.2790 (3)	0.0537 (11)	
N6	1.2968 (6)	0.4563 (4)	0.5530 (3)	0.0513 (11)	
N7	1.3579 (6)	0.5147 (3)	0.6166 (3)	0.0505 (10)	
N8	1.8445 (9)	0.4103 (5)	1.0418 (4)	0.0814 (16)	
O1	0.7156 (6)	−0.1358 (3)	−0.1262 (3)	0.0635 (11)	
O2	1.2202 (6)	0.5775 (3)	0.4444 (3)	0.0660 (11)	
O3	0.2036 (7)	0.2847 (3)	0.6588 (3)	0.0823 (14)	
O4	0.1417 (11)	0.1451 (4)	0.7472 (4)	0.120 (2)	
O5	0.4103 (8)	0.2082 (5)	0.7702 (4)	0.124 (2)	
S3	0.2325 (3)	0.22699 (11)	0.74187 (11)	0.0677 (5)	
F1	−0.0500 (8)	0.3171 (5)	0.8159 (4)	0.135 (2)	
F2	0.1803 (8)	0.3828 (3)	0.8371 (3)	0.1131 (17)	
F3	0.1446 (7)	0.2578 (3)	0.9181 (3)	0.0987 (14)	
C1	0.8156 (7)	0.0385 (4)	0.0907 (4)	0.0512 (13)	
H1	0.8508	0.0115	0.1513	0.061*	
C2	0.7630 (7)	−0.0201 (4)	0.0248 (4)	0.0498 (12)	
H2	0.7614	−0.0859	0.0398	0.060*	
C3	0.7118 (6)	0.0181 (4)	−0.0644 (3)	0.0425 (11)	
C4	0.7153 (9)	0.1136 (4)	−0.0820 (4)	0.0589 (15)	
H4	0.6811	0.1424	−0.1422	0.071*	
C5	0.7688 (9)	0.1674 (4)	−0.0114 (4)	0.0617 (15)	
H5	0.7693	0.2336	−0.0243	0.074*	
C6	0.6637 (7)	−0.0506 (4)	−0.1360 (4)	0.0471 (12)	
C7	0.4229 (7)	−0.0216 (4)	−0.3474 (4)	0.0524 (13)	
H7	0.4010	0.0463	−0.3488	0.063*	
C8	0.3555 (7)	−0.0722 (4)	−0.4237 (3)	0.0473 (12)	
C9	0.3767 (9)	−0.1707 (4)	−0.4261 (4)	0.0662 (16)	
H9	0.4402	−0.2080	−0.3765	0.079*	
C10	0.3077 (11)	−0.2158 (5)	−0.4991 (5)	0.082 (2)	
H10	0.3247	−0.2836	−0.4997	0.098*	
C11	0.2132 (10)	−0.1621 (5)	−0.5717 (5)	0.0746 (19)	
H11	0.1631	−0.1929	−0.6214	0.089*	
C12	0.1925 (8)	−0.0643 (4)	−0.5713 (4)	0.0559 (14)	
C13	0.2621 (7)	−0.0184 (4)	−0.4967 (3)	0.0484 (12)	
H13	0.2454	0.0494	−0.4962	0.058*	
C14	0.0978 (8)	−0.0051 (5)	−0.6452 (4)	0.0572 (14)	
C15	1.0095 (7)	0.4007 (4)	0.2630 (4)	0.0520 (13)	
H15	0.9526	0.4275	0.2064	0.062*	
C16	1.0737 (7)	0.4608 (4)	0.3233 (4)	0.0495 (12)	
H16	1.0617	0.5273	0.3079	0.059*	
C17	1.1562 (7)	0.4243 (4)	0.4069 (3)	0.0442 (11)	
C18	1.1695 (10)	0.3279 (4)	0.4243 (4)	0.0723 (19)	
H18	1.2229	0.2998	0.4813	0.087*	
C19	1.1061 (11)	0.2720 (4)	0.3597 (5)	0.078 (2)	
H19	1.1216	0.2048	0.3721	0.093*	
C20	1.2263 (7)	0.4947 (4)	0.4696 (4)	0.0477 (12)	
C21	1.4326 (7)	0.4693 (4)	0.6902 (4)	0.0510 (13)	
H21	1.4425	0.4016	0.6954	0.061*	
C22	1.5024 (7)	0.5212 (4)	0.7661 (4)	0.0490 (12)	
C23	1.4820 (8)	0.6210 (4)	0.7655 (4)	0.0575 (14)	
H23	1.4170	0.6576	0.7158	0.069*	
C24	1.5556 (9)	0.6667 (4)	0.8366 (4)	0.0648 (16)	
H24	1.5403	0.7345	0.8364	0.078*	
C25	1.6511 (8)	0.6137 (4)	0.9077 (4)	0.0615 (15)	
H25	1.7058	0.6451	0.9553	0.074*	
C26	1.6686 (7)	0.5153 (4)	0.9106 (4)	0.0535 (13)	
C27	1.5949 (7)	0.4690 (4)	0.8391 (4)	0.0504 (12)	
H27	1.6082	0.4012	0.8406	0.060*	
C28	1.7683 (8)	0.4570 (4)	0.9849 (4)	0.0581 (14)	
C29	0.1274 (11)	0.2994 (4)	0.8338 (5)	0.0696 (17)	
H28	1.287 (7)	0.397 (4)	0.575 (4)	0.047 (15)*	
H29	0.529 (8)	0.051 (4)	−0.217 (4)	0.055 (17)*	

### Atomic displacement parameters (Å^2^)

**Table:**

	*U*^11^	*U*^22^	*U*^33^	*U*^12^	*U*^13^	*U*^23^
Ag1	0.0727 (4)	0.0744 (4)	0.0628 (3)	−0.0187 (2)	−0.0167 (2)	−0.0289 (2)
N1	0.053 (3)	0.052 (3)	0.052 (3)	−0.012 (2)	−0.017 (2)	−0.010 (2)
N2	0.056 (3)	0.053 (3)	0.044 (2)	−0.004 (2)	−0.014 (2)	−0.015 (2)
N3	0.054 (3)	0.058 (3)	0.045 (2)	−0.010 (2)	−0.011 (2)	−0.017 (2)
N4	0.074 (4)	0.091 (4)	0.055 (3)	−0.024 (3)	−0.020 (3)	0.005 (3)
N5	0.064 (3)	0.052 (3)	0.046 (2)	−0.010 (2)	−0.015 (2)	−0.009 (2)
N6	0.062 (3)	0.053 (3)	0.041 (2)	−0.014 (2)	−0.018 (2)	−0.006 (2)
N7	0.051 (3)	0.060 (3)	0.042 (2)	−0.013 (2)	−0.0110 (19)	−0.009 (2)
N8	0.093 (4)	0.094 (4)	0.059 (3)	−0.016 (3)	−0.029 (3)	−0.005 (3)
O1	0.079 (3)	0.049 (2)	0.064 (2)	−0.013 (2)	−0.029 (2)	−0.0073 (18)
O2	0.090 (3)	0.051 (2)	0.059 (2)	−0.014 (2)	−0.028 (2)	−0.0045 (19)
O3	0.120 (4)	0.075 (3)	0.053 (3)	−0.018 (3)	−0.009 (2)	0.007 (2)
O4	0.200 (7)	0.061 (3)	0.108 (4)	−0.045 (4)	0.000 (4)	−0.006 (3)
O5	0.091 (4)	0.168 (6)	0.099 (4)	0.032 (4)	−0.022 (3)	−0.007 (4)
S3	0.0903 (12)	0.0504 (8)	0.0573 (9)	0.0049 (8)	−0.0142 (8)	0.0033 (7)
F1	0.096 (4)	0.161 (5)	0.134 (5)	0.022 (4)	0.016 (3)	0.014 (4)
F2	0.199 (6)	0.058 (2)	0.087 (3)	−0.033 (3)	0.010 (3)	−0.016 (2)
F3	0.163 (4)	0.074 (2)	0.055 (2)	−0.009 (3)	−0.002 (2)	0.0057 (19)
C1	0.053 (3)	0.061 (3)	0.040 (3)	−0.013 (3)	−0.015 (2)	0.000 (2)
C2	0.056 (3)	0.044 (3)	0.050 (3)	−0.012 (2)	−0.014 (2)	0.004 (2)
C3	0.039 (3)	0.050 (3)	0.040 (3)	−0.012 (2)	−0.011 (2)	−0.002 (2)
C4	0.082 (4)	0.049 (3)	0.046 (3)	−0.013 (3)	−0.027 (3)	0.004 (2)
C5	0.082 (4)	0.046 (3)	0.058 (3)	−0.014 (3)	−0.028 (3)	0.002 (3)
C6	0.045 (3)	0.052 (3)	0.045 (3)	−0.009 (2)	−0.014 (2)	−0.004 (2)
C7	0.054 (3)	0.058 (3)	0.044 (3)	−0.004 (3)	−0.013 (2)	−0.010 (2)
C8	0.047 (3)	0.056 (3)	0.040 (3)	−0.009 (2)	−0.010 (2)	−0.006 (2)
C9	0.079 (4)	0.055 (3)	0.066 (4)	−0.015 (3)	−0.029 (3)	0.000 (3)
C10	0.102 (5)	0.058 (4)	0.088 (5)	−0.016 (4)	−0.037 (4)	−0.009 (3)
C11	0.089 (5)	0.073 (4)	0.066 (4)	−0.023 (4)	−0.033 (3)	−0.013 (3)
C12	0.058 (3)	0.069 (4)	0.045 (3)	−0.021 (3)	−0.012 (2)	−0.008 (3)
C13	0.053 (3)	0.054 (3)	0.040 (3)	−0.011 (2)	−0.009 (2)	−0.008 (2)
C14	0.057 (3)	0.074 (4)	0.044 (3)	−0.023 (3)	−0.013 (3)	−0.004 (3)
C15	0.054 (3)	0.063 (3)	0.038 (3)	−0.004 (3)	−0.020 (2)	−0.009 (2)
C16	0.056 (3)	0.047 (3)	0.045 (3)	−0.005 (2)	−0.015 (2)	0.001 (2)
C17	0.049 (3)	0.054 (3)	0.029 (2)	−0.006 (2)	−0.006 (2)	−0.004 (2)
C18	0.115 (6)	0.051 (3)	0.050 (3)	−0.013 (3)	−0.037 (3)	0.006 (3)
C19	0.131 (6)	0.043 (3)	0.061 (4)	−0.017 (3)	−0.044 (4)	−0.001 (3)
C20	0.047 (3)	0.047 (3)	0.048 (3)	−0.004 (2)	−0.010 (2)	−0.008 (2)
C21	0.058 (3)	0.058 (3)	0.041 (3)	−0.018 (3)	−0.010 (2)	−0.005 (2)
C22	0.048 (3)	0.057 (3)	0.043 (3)	−0.013 (2)	−0.015 (2)	−0.003 (2)
C23	0.057 (3)	0.059 (3)	0.057 (3)	−0.010 (3)	−0.010 (3)	−0.001 (3)
C24	0.077 (4)	0.053 (3)	0.067 (4)	−0.015 (3)	−0.009 (3)	−0.012 (3)
C25	0.062 (4)	0.070 (4)	0.057 (3)	−0.019 (3)	−0.015 (3)	−0.019 (3)
C26	0.051 (3)	0.067 (4)	0.044 (3)	−0.012 (3)	−0.010 (2)	−0.011 (3)
C27	0.056 (3)	0.053 (3)	0.043 (3)	−0.012 (2)	−0.009 (2)	−0.006 (2)
C28	0.059 (4)	0.073 (4)	0.044 (3)	−0.012 (3)	−0.013 (3)	−0.009 (3)
C29	0.098 (5)	0.047 (3)	0.060 (4)	0.000 (3)	−0.015 (3)	0.008 (3)

### Geometric parameters (Å, °)

**Table:**

Ag1—N5	2.160 (4)	C7—H7	0.9500
Ag1—N1	2.169 (4)	C8—C9	1.381 (8)
N1—C1	1.333 (7)	C8—C13	1.390 (7)
N1—C5	1.335 (7)	C9—C10	1.379 (8)
N2—C6	1.354 (7)	C9—H9	0.9500
N2—N3	1.381 (6)	C10—C11	1.389 (9)
N2—H29	0.86 (6)	C10—H10	0.9500
N3—C7	1.278 (7)	C11—C12	1.370 (9)
N4—C14	1.140 (7)	C11—H11	0.9500
N5—C15	1.320 (7)	C12—C13	1.404 (7)
N5—C19	1.351 (8)	C12—C14	1.439 (8)
N6—C20	1.359 (7)	C13—H13	0.9500
N6—N7	1.371 (6)	C15—C16	1.366 (7)
N6—H28	0.90 (5)	C15—H15	0.9500
N7—C21	1.291 (7)	C16—C17	1.386 (7)
N8—C28	1.126 (8)	C16—H16	0.9500
O1—C6	1.215 (6)	C17—C18	1.368 (8)
O2—C20	1.207 (6)	C17—C20	1.513 (7)
O3—S3	1.407 (4)	C18—C19	1.365 (8)
O4—S3	1.432 (6)	C18—H18	0.9500
O5—S3	1.385 (6)	C19—H19	0.9500
S3—C29	1.798 (7)	C21—C22	1.467 (7)
F1—C29	1.347 (9)	C21—H21	0.9500
F2—C29	1.305 (8)	C22—C27	1.377 (7)
F3—C29	1.310 (7)	C22—C23	1.399 (8)
C1—C2	1.371 (7)	C23—C24	1.379 (8)
C1—H1	0.9500	C23—H23	0.9500
C2—C3	1.389 (7)	C24—C25	1.371 (9)
C2—H2	0.9500	C24—H24	0.9500
C3—C4	1.371 (7)	C25—C26	1.380 (8)
C3—C6	1.514 (7)	C25—H25	0.9500
C4—C5	1.377 (7)	C26—C27	1.389 (7)
C4—H4	0.9500	C26—C28	1.452 (8)
C5—H5	0.9500	C27—H27	0.9500
C7—C8	1.453 (7)		
			
N5—Ag1—N1	172.56 (17)	C11—C12—C13	120.4 (5)
C1—N1—C5	116.7 (4)	C11—C12—C14	122.0 (5)
C1—N1—Ag1	120.4 (3)	C13—C12—C14	117.6 (5)
C5—N1—Ag1	122.4 (4)	C8—C13—C12	119.9 (5)
C6—N2—N3	119.1 (5)	C8—C13—H13	120.0
C6—N2—H29	124 (4)	C12—C13—H13	120.0
N3—N2—H29	116 (4)	N4—C14—C12	177.3 (6)
C7—N3—N2	115.2 (5)	N5—C15—C16	123.6 (5)
C15—N5—C19	116.4 (5)	N5—C15—H15	118.2
C15—N5—Ag1	122.6 (3)	C16—C15—H15	118.2
C19—N5—Ag1	120.7 (4)	C15—C16—C17	119.8 (5)
C20—N6—N7	119.2 (5)	C15—C16—H16	120.1
C20—N6—H28	124 (4)	C17—C16—H16	120.1
N7—N6—H28	116 (4)	C18—C17—C16	117.0 (5)
C21—N7—N6	113.5 (4)	C18—C17—C20	126.1 (5)
O5—S3—O3	113.8 (4)	C16—C17—C20	116.9 (4)
O5—S3—O4	114.0 (4)	C19—C18—C17	120.0 (5)
O3—S3—O4	115.9 (3)	C19—C18—H18	120.0
O5—S3—C29	103.2 (4)	C17—C18—H18	120.0
O3—S3—C29	105.1 (3)	N5—C19—C18	123.1 (5)
O4—S3—C29	102.8 (4)	N5—C19—H19	118.4
N1—C1—C2	123.6 (5)	C18—C19—H19	118.4
N1—C1—H1	118.2	O2—C20—N6	124.5 (5)
C2—C1—H1	118.2	O2—C20—C17	120.9 (5)
C1—C2—C3	118.9 (5)	N6—C20—C17	114.5 (4)
C1—C2—H2	120.5	N7—C21—C22	120.6 (5)
C3—C2—H2	120.5	N7—C21—H21	119.7
C4—C3—C2	118.1 (5)	C22—C21—H21	119.7
C4—C3—C6	125.1 (4)	C27—C22—C23	119.4 (5)
C2—C3—C6	116.8 (4)	C27—C22—C21	118.2 (5)
C3—C4—C5	119.1 (5)	C23—C22—C21	122.4 (5)
C3—C4—H4	120.5	C24—C23—C22	120.3 (6)
C5—C4—H4	120.5	C24—C23—H23	119.8
N1—C5—C4	123.6 (5)	C22—C23—H23	119.8
N1—C5—H5	118.2	C25—C24—C23	119.7 (6)
C4—C5—H5	118.2	C25—C24—H24	120.2
O1—C6—N2	124.3 (5)	C23—C24—H24	120.2
O1—C6—C3	120.7 (4)	C24—C25—C26	120.7 (5)
N2—C6—C3	114.9 (4)	C24—C25—H25	119.7
N3—C7—C8	121.4 (5)	C26—C25—H25	119.7
N3—C7—H7	119.3	C25—C26—C27	119.8 (5)
C8—C7—H7	119.3	C25—C26—C28	122.2 (5)
C9—C8—C13	118.7 (5)	C27—C26—C28	118.0 (5)
C9—C8—C7	123.2 (5)	C22—C27—C26	120.1 (5)
C13—C8—C7	118.0 (5)	C22—C27—H27	120.0
C10—C9—C8	121.4 (6)	C26—C27—H27	120.0
C10—C9—H9	119.3	N8—C28—C26	178.7 (6)
C8—C9—H9	119.3	F2—C29—F3	108.7 (6)
C9—C10—C11	119.9 (6)	F2—C29—F1	106.0 (6)
C9—C10—H10	120.0	F3—C29—F1	106.0 (6)
C11—C10—H10	120.0	F2—C29—S3	113.5 (5)
C12—C11—C10	119.6 (5)	F3—C29—S3	113.9 (4)
C12—C11—H11	120.2	F1—C29—S3	108.1 (5)
C10—C11—H11	120.2		
			
C6—N2—N3—C7	−176.3 (5)	C15—C16—C17—C20	179.1 (5)
C20—N6—N7—C21	−175.6 (5)	C16—C17—C18—C19	1.3 (10)
C5—N1—C1—C2	0.4 (8)	C20—C17—C18—C19	−177.5 (6)
Ag1—N1—C1—C2	−172.1 (4)	C15—N5—C19—C18	2.4 (11)
N1—C1—C2—C3	0.3 (8)	Ag1—N5—C19—C18	176.9 (6)
C1—C2—C3—C4	−0.5 (8)	C17—C18—C19—N5	−2.7 (12)
C1—C2—C3—C6	177.1 (5)	N7—N6—C20—O2	3.5 (8)
C2—C3—C4—C5	0.1 (9)	N7—N6—C20—C17	−177.5 (4)
C6—C3—C4—C5	−177.3 (5)	C18—C17—C20—O2	174.2 (6)
C1—N1—C5—C4	−0.9 (9)	C16—C17—C20—O2	−4.5 (8)
Ag1—N1—C5—C4	171.4 (5)	C18—C17—C20—N6	−4.8 (8)
C3—C4—C5—N1	0.7 (10)	C16—C17—C20—N6	176.4 (5)
N3—N2—C6—O1	−0.2 (8)	N6—N7—C21—C22	−178.9 (5)
N3—N2—C6—C3	179.8 (4)	N7—C21—C22—C27	−174.4 (5)
C4—C3—C6—O1	156.7 (6)	N7—C21—C22—C23	4.1 (8)
C2—C3—C6—O1	−20.7 (8)	C27—C22—C23—C24	0.8 (9)
C4—C3—C6—N2	−23.3 (8)	C21—C22—C23—C24	−177.6 (5)
C2—C3—C6—N2	159.3 (5)	C22—C23—C24—C25	0.8 (9)
N2—N3—C7—C8	−177.8 (5)	C23—C24—C25—C26	−2.4 (10)
N3—C7—C8—C9	1.9 (9)	C24—C25—C26—C27	2.5 (9)
N3—C7—C8—C13	−179.5 (5)	C24—C25—C26—C28	−179.8 (6)
C13—C8—C9—C10	0.1 (10)	C23—C22—C27—C26	−0.8 (8)
C7—C8—C9—C10	178.7 (6)	C21—C22—C27—C26	177.7 (5)
C8—C9—C10—C11	−0.6 (12)	C25—C26—C27—C22	−0.9 (8)
C9—C10—C11—C12	1.4 (12)	C28—C26—C27—C22	−178.6 (5)
C10—C11—C12—C13	−1.7 (10)	O5—S3—C29—F2	66.4 (6)
C10—C11—C12—C14	179.2 (7)	O3—S3—C29—F2	−53.2 (6)
C9—C8—C13—C12	−0.4 (8)	O4—S3—C29—F2	−174.8 (5)
C7—C8—C13—C12	−179.1 (5)	O5—S3—C29—F3	−58.8 (7)
C11—C12—C13—C8	1.2 (9)	O3—S3—C29—F3	−178.3 (5)
C14—C12—C13—C8	−179.6 (5)	O4—S3—C29—F3	60.0 (7)
C19—N5—C15—C16	−0.8 (9)	O5—S3—C29—F1	−176.3 (5)
Ag1—N5—C15—C16	−175.2 (4)	O3—S3—C29—F1	64.1 (6)
N5—C15—C16—C17	−0.5 (9)	O4—S3—C29—F1	−57.6 (6)
C15—C16—C17—C18	0.3 (8)		

### Hydrogen-bond geometry (Å, °)

**Table:**

*D*—H···*A*	*D*—H	H···*A*	*D*···*A*	*D*—H···*A*
N2—H29···O5^i^	0.86 (6)	2.27 (6)	3.125 (9)	173 (5)
N6—H28···O3^ii^	0.90 (5)	2.12 (6)	2.982 (7)	161 (5)

Symmetry codes: (i) *x*, *y*, *z*−1; (ii) *x*+1, *y*, *z*.
